# Correlation of Visual Recovery and Increased Ellipsoid Zone Reflectivity After Successful Macular Hole Surgery

**DOI:** 10.4274/tjo.galenos.2020.21456

**Published:** 2020-10-30

**Authors:** Büşra Yılmaz Tuğan, Levent Karabaş, Fatih Yenihayat, Sevgi Subaşı, Enes Kesim, Berna Özkan

**Affiliations:** 1 Kocaeli University Faculty of Medicine, Department of Ophthalmology, Kocaeli, Turkey; 2 Dünyagöz Kocaeli Hospital, Clinic of Ophthalmology, Kocaeli, Turkey; 3 Tuzla State Hospital, Clinic of Ophthalmology, İstanbul, Turkey; 4 Acıbadem Mehmet Ali Aydınlar University Faculty of Medicine, Department of Ophthalmology, İstanbul, Turkey

**Keywords:** Absolute and relative reflectivity, ellipsoid zone reflectivity, macular hole, vitrectomy

## Abstract

**Objectives:**

To assess changes in reflectivity of the retinal pigment epithelium (RPE), ellipsoid zone (EZ), and external limiting membrane (ELM) on spectral domain-optical coherence tomography (SD-OCT) images and the effects of reflectivity changes on visual acuity improvement after vitrectomy in macular hole patients.

**Materials and Methods:**

Twenty-four eyes of 24 patients with idiopathic full-thickness macular hole closed after vitrectomy were retrospectively reviewed. The “plot profile” function of the medical imaging software was used by a single masked physician to analyze RPE, EZ, and ELM reflectivity on OCT images at postoperative 1 month and 12 months.

**Results:**

Absolute and relative EZ reflectivity showed highly significant increases at postoperative 12 months compared to 1 month (p<0.001 and p<0.001, respectively). Absolute and relative EZ reflectivity changes from postoperative month 1 to month 12 after macular hole surgery were significantly correlated with best corrected visual acuity improvement (p=0.012 and p=0.020, respectively).

**Conclusion:**

EZ reflectivity can be a predictor of functional and anatomical improvement after macular hole surgery.

## Introduction

Idiopathic macular hole (MH) is a full-thickness anatomical defect of the neural retina at the fovea that can lead to central vision loss. Edema and detachment of the sensory retina may lead to the progression of hole enlargement, retinal pigment epithelium (RPE) atrophy, and decrease in visual acuity.^1^

Kelly and Wendel^2^ first introduced vitrectomy and intraocular gas tamponade for MH surgery. Later, with progress in surgical instrumentation and techniques, anatomical closure rates reached over 90%.^3^ Large numbers of associated factors lead to variation in visual outcomes despite anatomical closure. Recent improvements in the resolution of optical coherence tomography (OCT) have enabled ophthalmologists to observe retinal structures more precisely. Researchers have suggested that maximal visual recovery may take a year or more and is linked to photoreceptor layer status.^4,5,6,7^ Furthermore, recent studies using spectral-domain OCT (SD-OCT) support the suggestion that delayed visual recovery may be related to the reorganization of the photoreceptor layer.^8,9^

Current literature emphasizes that structural or functional impairment in related retinal layers causes lower reflectivity.^10,11,12^

In this study, we intended to observe the effects of vitrectomy on RPE, ellipsoid zone (EZ), and external limiting membrane (ELM) reflectivity using image processing computer software (ImageJ 1.47v, Wayne Rasband, National Institutes of Health, USA, http://imagej.nih.gov/ij) in MH patients.^13^ In addition, we analyzed the association between visual recovery and retinal layer integrity and reflectivity after vitrectomy.

## Materials and Methods

Twenty-four eyes of 24 patients with idiopathic full-thickness MH closed after vitrectomy between January 2015 and June 2016 were included in this retrospective study. The local ethics committee approved this study, and we followed the tenets of the Declaration of Helsinki. All patients were diagnosed with stage 2 or 3 idiopathic MH according to Gass classification system and were followed for at least 12 months postoperatively.^14^ Patients with full-thickness MH that closed successfully after vitrectomy confirmed by SD-OCT examinations (Spectralis OCT; Heidelberg Engineering, Heidelberg, Germany) were included. Patients with previous retinal surgery, macular degeneration, diabetic retinopathy, inflammatory ocular diseases, retinal vascular occlusions, hypertensive retinopathy, MH associated with other pathology, lamellar MH, or pseudo-MH were excluded. Reactive gliosis (glial healing) is a complex process that is considered to promote retinal repair following pathological insult or surgery. Therefore, we excluded patients showing glial healing in 2 or more layers, which may result in inability to differentiate between layers in OCT images.

All patients underwent 23-gauge pars plana vitrectomy and internal limiting membrane (ILM) peeling with forceps in an area of 2-3 optic disc diameters around the MH. Sulfur hexafluoride (SF6) or perfluoropropane (C3F8) gas tamponade were used and patients were informed to remain in a face-down position for at least 3 days after surgery. Cataract surgery was not performed at the time of vitrectomy in any patient.

**Figure 1:**
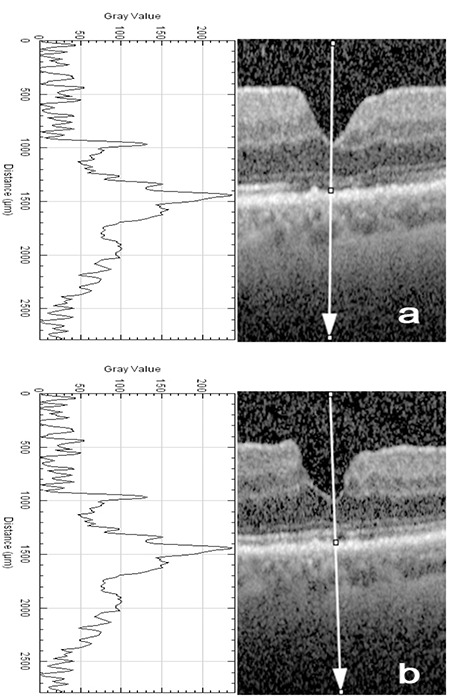
Peaks of the retinal pigment epithelium (RPE), ellipsoid zone (EZ), and external limiting membrane (ELM) on reflectivity graph (left) and gray-scale optical coherence tomography images (right) from postoperative 1 month (a) and 12 months (b) obtained from an image processing program (ImageJ). ImageJ gives reflectivity values along a line (vertical white arrow) and creates reflectivity graph

Visual acuity was measured and SD-OCT was performed the day before MH surgery and 1 month and 12 months postoperatively. Best corrected visual acuity (BCVA) was converted to the logarithm of the minimal angle of resolution (logMAR) equivalent. SD-OCT images were exported to the Java-based image processing computer software, ImageJ.^13,15^ ImageJ is a reliable tool with high inter- and intra-observer reproducibility and has been used in several recent studies in the field of ophthalmology.^10,16,17,18^ The “plot profile” function of ImageJ was used by a single masked physician (F.Y.) to analyze OCT images (Figure 1).^10,13,15^ A vertical straight line passing through the center of the fovea was drawn from the vitreous cavity to the choroid to obtain reflectivity graph and reflectivity values along the line (Figure 1).^10,15^ In a normal OCT image, the histologic order of reflectivity is RPE layer, EZ (formerly called the photoreceptor inner segment/outer segment [IS/OS] junction), and ELM, respectively. In studies regarding reflectivity of retina, the outermost highly reflective band is thought to represent RPE^19,20^, so the highest value was accepted as the reflectivity of the RPE layer, and relative reflectivity of the EZ or ELM was calculated by dividing EZ or ELM reflectivity by RPE reflectivity according to this formula:

Relative reflectivity (arbitrary unit) = (reflectivity of EZ or ELM)/(reflectivity of RPE) x100.^10^

### Statistical Analysis

All statistical analyses were performed using IBM SPSS for Windows version 20.0 (IBM Corp., Armonk, NY, USA). The normality of data distribution was tested with Kolmogorov-Smirnov test. Continuous variables were expressed as mean ± standard deviation (SD) and categorical variables as counts and percentages. The significance of differences between the time points was analyzed using paired-samples t-test for normally distributed variables and Wilcoxon signed-rank test for nonnormally distributed variables. Pearson and Spearman correlation analysis was used to determine associations between continuous variables. A two-sided p value <0.05 was considered statistically significant.

## Results

Twenty-four eyes of 24 consecutive idiopathic full-thickness MH patients (14 male, 10 female) with a mean age of 64.46±10.90 years were included in the study (Table 1). The MHs were classified as stage 2 in 13 eyes and stage 3 in 11 eyes (Table 1). The mean BCVA (logMAR ± SD) was 0.52±0.17 before surgery and increased to 0.35±0.15 at postoperative 12 months (p<0.001). The mean BCVA at postoperative 1 month was 0.51±0.22 and increased to 0.35±0.15 at postoperative 12 months (p=0.006) (Table 1). Eyes with any findings of exhibited glial healing in the RPE, EZ, and ELM layer excluded from statistical analysis (1 patient, 6 patients, and 3 patients, respectively).

In this retrospective study, we performed image analysis and determined the reflectivity of RPE, EZ, and ELM of all subjects. Absolute and relative EZ reflectivity showed a highly significant increase at postoperative 12 months compared to 1 month (p<0.001 and p<0.001, respectively) (Table 2). However, there were no differences between RPE and ELM reflectivities at postoperative 1 month and 12 months.

In addition, changes in absolute and relative reflectivity parameters according to BCVA improvement were analyzed (Table 3). The changes in absolute and relative EZ reflectivity from postoperative 1 month to 12 months after MH surgery were correlated with the change in BCVA from preoperative to postoperative 12 months (p=0.012 and p=0.020, respectively). However, changes in absolute and relative ELM reflectivity from postoperative 1 month to 12 months were not correlated with change in BCVA from preoperative to postoperative 12 months (p=0.337 and p=0.573). Change in absolute RPE reflectivity from postoperative 1 month to 12 months was not correlated with the pre- to postoperative change in BCVA (p=0.369). Changes in absolute and relative reflectivity measurements from postoperative 1 month to 12 months were not correlated with BCVA improvement from postoperative 1 month to 12 months. Absolute and relative reflectivity measurements at postoperative 1 month were not correlated with BCVA at postoperative 12 months.

Preoperative MH diameter was not correlated with absolute or relative reflectivities at postoperative 1 month and 12 months or with the changes in reflectivity from postoperative 1 month to 12 months. In the grade 2 and 3 MH patient groups, only absolute and relative EZ reflectivities showed a statistically significant increase from postoperative 1 month to 12 months (p=0.017 and p=0.003, respectively). Absolute RPE reflectivity and absolute and relative ELM reflectivities did not show significant differences between postoperative 1 month and 12 months in two groups (p=0.855, p=0.431 and p=0.439, respectively).

## Discussion

Idiopathic MH is a pathological condition that causes disruption of the retinal layers alignment. The EZ, which signifies the photoreceptor inner segment ellipsoid with densely packed mitochondria^21^, reflects photoreceptor integrity and function and is seen as a highly reflective continuous band just above the RPE in ultra-high-resolution OCT.^22^ In recent years, some authors have emphasized EZ integrity as a prognostic factor for the increase in visual acuity after vitrectomy in some retinal diseases.^8,23,24,25,26,27,28,29^ Baba et al.^7^ showed the importance of normal EZ for visual improvement after MH surgery. In a study by Shimozono et al.^30^ including 30 eyes of 30 patients with idiopathic MH that underwent successful vitrectomy, photoreceptor OS restoration was described as an important factor for visual recovery after MH surgery. Michalewska et al.^31^ revealed resolved photoreceptor layer defects at postoperative 12 months in 70.5% of MH surgery patients. In addition, Kim et al.^32^ used a photoreceptor layer map to show gradually decreased hyporeflectivity with improvement in visual acuity after MH surgery.

In the current study, we measured the reflectivity of the RPE, EZ, and ELM on SD-OCT scans using ImageJ image processing software to understand the effects of vitrectomy on the functionality of these layers in MH patients. We observed significant increases in EZ reflectivity (both absolute and relative) at postoperative 12 months compared to postoperative 1 month, whereas RPE and ELM reflectivity did not show a difference between postoperative 1 month and 12 months. Some studies showed that decreased EZ reflectivity is associated with poor photoreceptor function.^19,20^ Our results also showed a significant positive correlation between EZ reflectivity and BCVA improvement.

Schumann et al.^33^ reviewed patients with lamellar MH and macular pseudohole according to EZ and ELM integrity or discontinuity. They postulated that integrity of the ELM appeared to be more critical for visual improvement than integrity of the EZ. Chang et al.^34^ retrospectively reviewed 60 eyes of 56 patients that underwent successful vitrectomy and ILM peeling for idiopathic MH and concluded that postoperative visual acuity was correlated with restored ELM and EZ line. Furthermore, in a retrospective study, eyes with both ELM and EZ disruption showed significantly lower BCVA measurements at postoperative 6 weeks than those with only EZ disruption, suggesting that ELM integrity is a critical factor for photoreceptor layer healing and visual improvement.^35^ However, in the present study we did not find any correlation between RPE or ELM reflectivity and visual acuity improvement.

Other studies also showed that recovery of the macular contour, ELM, and EZ affected the recovery of vision after MH surgery.^36,37^ Kim et al.^38^ reported that the EZ recovered postoperatively in 19 patients (73.1%) and that better preoperative BCVA, smaller basal hole diameter, and shorter axial length were observed in eyes with recovered EZ. In another study, smaller defects in the EZ and absence of an ELM defect were found to be associated with better postoperative BCVA.^39^

### Study Limitations

This study has some limitations, including the small sample size and its retrospective, non-randomized design. Potential transmission artifacts or some degrees of gliosis could influence the results of the current study.

## Conclusion

In conclusion, EZ reflectivity seems to be essential for visual function and may be a predictor of functional and anatomical improvement after vitrectomy in MH patients.

## Figures and Tables

**Table 1:**
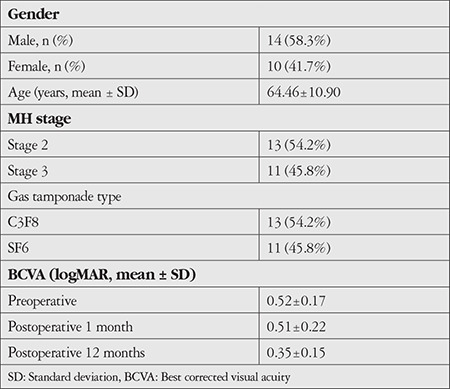
Demographic data of macular hole (MH) patients (n=24)

**Table 2:**
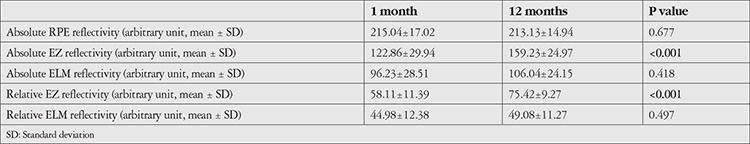
Comparison of retinal pigment epithelium (RPE), ellipsoid zone (EZ), and external limiting membrane (ELM) reflectivities at postoperative 1 and 12 months

**Table 3:**
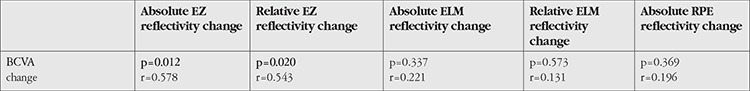
Correlation of preoperative to postoperative month-12 BCVA changes with retinal pigment epithelium (RPE), ellipsoid zone (EZ), and external limiting membrane (ELM) reflectivity from postoperative month 1 to month 12
